# Inflammation and oxidative stress in the sputum of patients with cystic fibrosis: correlations with lung function, aerobic fitness, and morbidity

**DOI:** 10.1590/1984-0462/2026/44/2025147

**Published:** 2026-06-22

**Authors:** Denise Lautenschleger Fischer, Fernanda Maria Vendrusculo, Mariana Severo da Costa, Maria Claudia Rosa Garcia, Jarbas Rodrigues de Oliveira, Márcio Vinícius Fagundes Donadio

**Affiliations:** a Pontifícia Universidade Católica do Rio Grande do Sul, Porto Alegre, RS, Brazil.; b Universitat Internacional de Catalunya, Barcelona, Spain.

**Keywords:** Cystic fibrosis, Inflammation, Oxidative stress, Pulmonary function, Aerobic fitness, Fibrose cística, Inflamação, Estresse oxidativo, Função pulmonar, Aptidão aeróbica

## Abstract

**Objective:**

The aim of this study was to compare the sputum inflammatory profile of children and adolescents with cystic fibrosis (CF) to that of healthy saliva controls and to assess the association between these markers and key clinical variables.

**Methods:**

This is an observational study comparing a group of CF patients with a healthy control group, including sputum samples from patients aged 6–18 years, followed up at a CF referral center. All procedures were performed on the same day, including spirometry, cardiopulmonary exercise testing, and sputum collection and processing. Demographic and clinical data were collected from patient records.

**Results:**

A total of 12 CF patients were included with a mean age of 13.0±2.8 years, a mean forced expiratory volume in one second of 86.4±20.8%, and a peak oxygen consumption (VO_2_peak) of 35.4±4.6 mL kg ^−1^ min^−1^. A predominance of neutrophils was found in the sputum. The median (interquartile range) of interleukin-8 (IL-8) was 386.8 (310.5) pg/g of protein, and thiobarbituric acid-reactive substances (TBARS) had a median of 2.2 (0.9) μM/g of protein. When comparing CF patients and healthy individuals, the CF group showed higher IL-8 concentrations (p<0.001) and increased TBARS levels (p=0.01). A correlation was found between IL-6 concentration and the number of days on antibiotics (rho=0.71; p=0.009), and an inverse moderate correlation was found between IL-8 and forced expiratory flow between 25 and 75% (FEF_25–75%_) (rho=-0.62; p=0.033).

**Conclusions:**

Individuals with CF exhibited increased sputum inflammation compared with healthy controls, which correlated with lung function and morbidity but not with aerobic fitness.

## RESUMO

**Objetivo:**

Comparar o perfil inflamatório do escarro de crianças e adolescentes com fibrose cística (FC) com saliva de controles saudáveis e avaliar a associação desses marcadores com variáveis clínicas importantes.

**Métodos:**

Este é um estudo observacional que compara pacientes com FC a um grupo controle saudável, incluindo amostras de escarro de pacientes com idades entre seis e 18 anos, acompanhados em um centro de referência para FC. Todos os procedimentos foram realizados no mesmo dia, incluindo espirometria, teste de exercício cardiopulmonar (TECP), coleta e processamento do escarro. Dados demográficos e clínicos foram coletados dos prontuários dos pacientes.

**Resultados:**

Foram incluídos 12 pacientes com FC, com idade média de 13,0±2,8 anos, 86,4±20,8% do volume expiratório forçado no primeiro segundo (VEF1) e 35,4 ± 4,6 mL.kg^-1^ .min^-1^ de consumo de oxigênio de pico (VO_2_pico). Foi observada predominância de neutrófilos no escarro. A mediana (intervalo interquartil — IQR) de IL-8 foi de 386,8 (310,5) pg/g de proteína, e a TBARS apresentou mediana de 2,2 (0,9) μM/g de proteína. Ao comparar pacientes com FC e a saliva controle de indivíduos saudáveis, observaram-se concentrações mais elevadas (p<0,001) de IL-8 e aumento na TBARS (p=0,01) no grupo com FC. Foi encontrada uma correlação entre a concentração de IL-6 e o número de dias em uso de antibióticos (rho=0,71; p=0,009) e uma correlação inversa moderada entre IL-8 e FEF_25-75%_ (rho=-0,62; p=0,033).

**Conclusões:**

Indivíduos com FC apresentaram aumento da inflamação do escarro em comparação com controles saudáveis, o que se correlaciona com a função pulmonar e a morbidade, mas não com a aptidão aeróbica.

## INTRODUCTION

 Cystic fibrosis (CF) is characterized by a mutation of the gene that encodes the CFTR protein (cystic fibrosis transmembrane conductance regulator). Alterations in the exocrine epithelial mechanism lead to intracellular chloride accumulation, which, combined with increased sodium absorption, increases mucus production and viscosity. As a result, mucociliary clearance is impaired, and airflow is reduced. These factors, associated with inflammation and infection, contribute to structural damage in the airways.^1^

 A pro-inflammatory state may be present in the early phase of the disease, facilitating pathogen colonization.^2^ Subsequently, sustained inflammation contributes to the ongoing influx of neutrophils and the release of pro-inflammatory cytokines, such as interleukin-8 (IL-8).^3^ Another characteristic is the release of thiobarbituric acid-reactive substances (TBARS), which, when elevated, disrupt antioxidant mechanisms and increase oxidative stress-induced cellular damage.^4^

 The occurrence of exacerbations is associated with the expansion or modification of the pulmonary microbiome, which also contributes to a greater inflammatory response,^5-7^ potentially impacting the progression of lung disease. Evidence shows that both exercise capacity, measured by peak oxygen consumption (VO_2_peak) via cardiopulmonary exercise testing (CPET), and lung function, assessed by forced expiratory volume in one second (FEV_1_) and forced expiratory flow between 25 and 75% of forced vital capacity (FEF_25–75%_), are important predictors of morbidity and mortality.^8^ However, evidence on the basal inflammatory profile of sputum from non-exacerbated CF patients and the possible associations between inflammation, oxidative stress, exercise capacity, and lung function has been limited so far. 

 This study aimed to compare the inflammatory profile of sputum from children and adolescents with CF with that of saliva from healthy individuals. Additionally, this study aimed to assess the association of these markers with key clinical variables (lung function, aerobic fitness, and morbidity markers) in children and adolescents with CF. 

## METHOD

 This is an analytical, observational study comparing a group of CF patients with a healthy control group. Sputum samples were collected from children and adolescents in regular follow-up at a CF referral center. Inclusion criteria were ages 6–18 years and a genetic diagnosis of CF. Individuals with motor or cognitive impairments that could interfere with the proposed evaluations, as well as patients with clinical symptoms requiring hospitalization or exacerbated on the day of testing, were excluded. Saliva samples from healthy individuals (no diagnosis of chronic diseases or active acute symptoms that could potentially influence the measurements) within the same age range were obtained from a control sample bank in our laboratory and used as a non-invasive comparator for sputum inflammatory and oxidative stress profiles measured in CF patients. Saliva has been shown to contain measurable inflammatory markers relevant to respiratory disease and to correlate with airway biomarkers in several studies; moreover, parallel analyses indicate that salivary contamination does not substantially bias most sputum inflammatory measurements when samples are properly processed.^9^ Therefore, saliva from healthy individuals provides a pragmatic and validated comparator for baseline inflammatory levels. 

 Experiments and data collection were conducted from August 2022 to November 2023. On the same day, the following procedures were performed: spirometry, CPET, sputum collection (spontaneous or induced), and processing of sputum samples. Demographic and clinical data were collected directly from patient records. The University Research Ethics Committee approved the study under CAAE number 60097222.6.0000.5336, and all legal guardians and/or participants signed a consent form before inclusion. 

 Sample size calculation was performed based on the IL-8 variable, considering a standard deviation of 37 ng/mL,^10^ an alpha error of 5%, a power of 90%, and a two-tailed test. The estimate was made to detect a minimum difference of 60 ng/mL between patients with CF and healthy controls, using a 1:2 control-to-CF group ratio, requiring 12 sputum samples from patients with CF. The calculation was performed using the online tool Granmo 8.0 (REGICOR, IMIM, Barcelona). 

 The sputum samples were either voluntarily expectorated or induced using the protocol adapted from Pizzichini et al.^11^ with 3% saline inhalation. The sputum sample was macroscopically separated from saliva and weighed (150–250 mg) in a Falcon tube. Four times the sample volume of dithiothreitol and Dulbecco’s phosphate-buffered saline was added. The mixture was passed through a nylon filter and centrifuged at 5,000 rpm for 5 min. Histological slides were prepared using a cytocentrifuge and stained with the Fast Panoptic method (Laborclin), and differential cell counts were expressed as percentages for each cell type. The resulting supernatant was divided into treated and untreated portions and frozen at −20^°^C. 

 Proteases (Matrix metallopeptidase 2 MMP-2) were measured in the untreated supernatant. At the same time, IL-17A, IL-6, IL-8, and IL-10 were quantified in supernatants treated with protease inhibitors (30 μL of ethylenediaminetetraacetic acid and 15 μL of phenylmethylsulfonyl fluoride) using a specific reagent kit for the MagPix platform (MILLIPLEX^®^), following the manufacturer’s instructions. Data were analyzed using the xPONENT^®^ version 4.2 software (MILLIPLEX^®^) and were expressed in pg/gram of protein. TBARS concentrations were also quantified in the supernatant. For this, 10 μL of sputum supernatant was mixed with 10 μL of sodium dodecyl sulfate (12.4 mM) and 400 μL of thiobarbituric acid. After heating for 60 min, the mixture was centrifuged at 750g for 10 min at 25^°^C. The reaction between thiobarbituric acid and malondialdehyde was measured using a spectrophotometer (532 nm, Genesis 8/Spectronic), with values expressed in μM/g of protein. 

 The following data were collected from individual electronic records: age, sex, ethnicity, height, weight, body mass index (BMI), genetic mutation status, and airway bacterial colonization. Additionally, the number of hospitalizations and the number of days on oral and/or intravenous antibiotics during the year before study inclusion were recorded. 

 Spirometry was performed using the KoKo spirometer (Louisville, CO, USA). The subjects were instructed to take a maximum inhalation followed by a maximum exhalation lasting at least three seconds. The acceptability and reproducibility criteria followed the guidelines set by the American Thoracic Society/European Respiratory Society.^12^ The following parameters, measured in liters and z-scores, were recorded: forced vital capacity (FVC), FEV_1_, FEV_1_/FVC ratio, and FEF_25–75%_. 

 To assess aerobic fitness, an adapted protocol from a previous study was used,^13^ following the recommendations of the American College of Sports Medicine,^14^ and conducted by trained researchers. An ergospirometry system with a gas analyzer (VO2000; Medical Graphics Corp., St. Paul, MN, USA) was employed. The test consisted of a ramp protocol, with two minutes of familiarization on the treadmill at 3 km/h without incline. Then, the treadmill incline was increased by 3%, and the speed was gradually increased by 0.5 km/h every minute until the test was completed. 

 The following parameters were assessed: heart rate (HR), peripheral oxygen saturation (SpO_2_), peak oxygen consumption (VO_2_peak), minute ventilation (V_E_), ventilatory equivalent for oxygen consumption (V_E_ /VO_2_), ventilatory equivalent for carbon dioxide production (V_E_ /VCO_2_), respiratory exchange ratio (RER), and breathing reserve (BR). The ventilatory threshold (VT_1_) was defined as the point where there was an increase in V_E_/VO_2_ without a corresponding increase in V_E_ /VCO_2_. BR was calculated as the difference between the estimated maximum voluntary ventilation at rest and VE at peak exercise, and was expressed as a percentage (%). The test was considered maximal and acceptable when patients met at least three of the following criteria: visible exhaustion; RER >1.05; maximum HR >180 bpm; and/or a plateau in oxygen consumption. 

 Data normality was assessed using the Shapiro-Wilk test. Continuous data with a parametric distribution were expressed as mean and standard deviation, while non-parametric data were presented as median and interquartile range (IQR). Categorical variables were presented as absolute and relative frequencies. Comparisons between variables were performed using the Mann-Whitney test. Correlations were evaluated using the Spearman correlation test, with the Rho coefficient presented. Correlations were classified as weak (<0.4), moderate (0.4–0.7), or strong (>0.7). All analyses and data processing were conducted using SPSS version 18.0. The significance level adopted was 5% (p<0.05). 

## RESULTS

 Sputum samples were obtained from 12 CF patients. The mean age of the individuals was 13.0±2.8 years, with 66.7% being male. Approximately 58.3% of the subjects were heterozygous for the F508del mutation, 16.7% were chronically colonized by *Pseudomonas aeruginosa*, and none were on any CFTR modulator therapy during the study period. Regarding morbidity, the average duration of antibiotic therapy was 91.1±99.7 days. Only two patients had hospitalizations during this period. The mean FEV_1_ (z-score) was 1.3±1.8, indicating a sample with mild-to-moderate impairment. Complete sample characterization data are presented in Table 1. 

**Table 1.** Main characteristics of cystic fibrosis patients.

Variables		n=12
Demographics		
	Age (years)	13.0 (2.8)
	Male, n (%)	8 (66.7)
Anthropometrics		
	Weight (kg)	42.4±11.3
	Height (cm)	150.1±13.3
	BMI (kg/m^2^)	18.5±2.6
	BMI (z-score)	-0.1±0.9
Clinical		
	Pancreatic insufficiency, n (%)	12 (100)
	Chronic Pseudomonas aeruginosa, n (%)	2 (16.7)
	Days on antibiotic use	91.1±99.7
	Hospitalizations, n (%)	2 (16.7)
Genotyping		
	F508del homozygous, n (%)	7 (58.3)
	F508del heterozygous, n (%)	4 (33.3)
	Other mutations, n (%)	1 (8.3)
Lung function		
	FEV_1_ (% predicted)	86.4±20.8
	FEV_1_ (z-score)	-1.3±1.8
	FVC (% predicted)	92.5±20.2
	FVC (z-score)	-0.7±1.7
	FEV_1_/FVC (% predicted)	91.3±7.6
	FEV_1_/FVC (z-score)	-1.1±0.9
	FEF_25–75%_ (% predicted)	75.5±33.7
	FEF_25–75%_ (z-score)	-1.3±1.7

Kg: kilograms; cm: centimeters; m: meters; BMI: body mass index; FEV _1_: forced expiratory volume in the first second; FVC: forced vital capacity; FEF_25–75%_: forced expiratory flow between 25 and 75% of vital capacity. Notes: Values expressed as mean±standard deviation or absolute (relative) frequency

**Figure 1:**
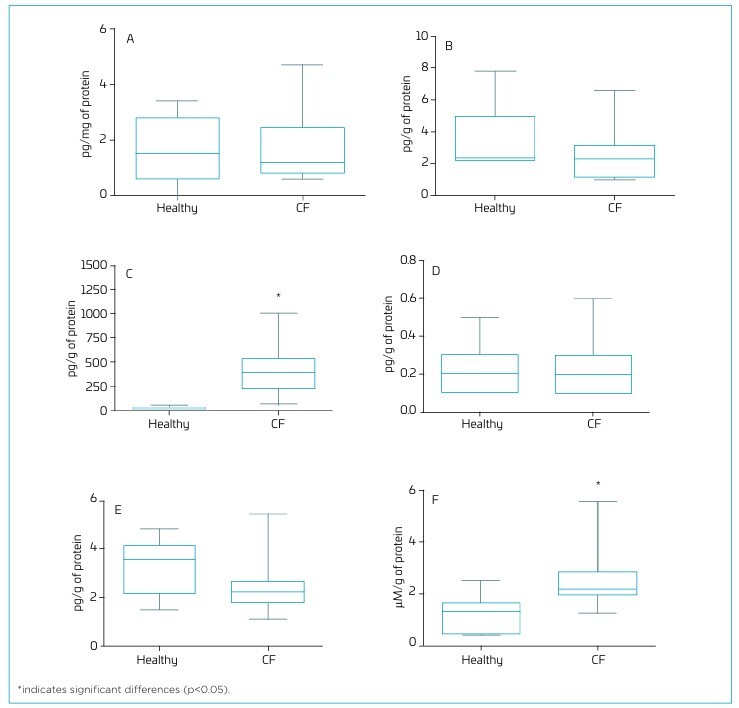
Comparison of inflammatory and oxidative stress markers between cystic fibrosis (CF) and healthy individuals. The graphs compare individuals with CF (n=12) and healthy controls (n=7) and show their respective concentrations of IL-17A (A), IL-6 (B), IL-8 (C), IL-10 (D), MMP-2 (E), and TBARS (F). Data presented as median, interquartile range, and total range, and compared using the Mann-Whitney test.

 Regarding sputum samples, about 75% were spontaneously expectorated. A predominance of neutrophils was found in the differential count, representing 82.7±5.1% of all counted cells (Table 2). Regarding the inflammatory and oxidative stress profile, the median (IQR) of IL-8 was 386.8 (310.5) pg/g of protein, and cellular damage, as measured by TBARS, had a median (IQR) of 2.2 (0.9) of protein. Complete data on the inflammatory profile, metalloproteinase, and oxidative stress are presented in Table 3. When comparing the concentrations of interleukins, MMP-2, and TBARS between CF patients and healthy individuals, higher concentrations (p<0.001) of IL-8 were observed in the CF group (Figure 1C). Additionally, Figure 1F shows higher TBARS concentrations in the CF group than in healthy ndividuals (p=0.01). No significant differences were found for the other variables (Figure 1A, B, D, and E ). 

**Table 2.** Cell characterization of sputum samples.

Variables		n=12
Sputum collection		
	Spontaneous, n (%)	9 (75.0)
	Weight of portion (mg)	200.7 (42.9)
	Weight of portion (mg)	200.7 (42.9)
Total cell count		
	Total count	294.8±202.3
	Absolute number (× 10^4^ cells/mL)	5.2±3.5
	Viability (%)	95.5±3.2
Differential cell count		
	Neutrophils (%)	82.7±5.1
	Macrophages (%)	12.8±3.9
	Lymphocytes (%)	4.0±2.3
	Eosinophils (%)	0.4±0.5

mg: milligrams; mL: milliliters. Notes: Values expressed in mean±standard deviation or absolute (relative) frequency, as indicated.

**Table 3.** Measurements of cytokines, metalloproteinase, and oxidative stress in sputum samples of cystic fibrosis children and adolescents.

Variables		n=12
Cytokines		
	IL-17A, pg/g protein	1.2 (1.6)
	IL-6, pg/g protein	2.3 (2.0)
	IL-8, pg/g protein	386.8 (310.5)
	IL-10, pg/g protein	0.2 (0.2)
Matrix metalloproteinases		
	MMP-2, pg/g protein	2.2 (0.9)
Oxidative stress		
	TBARS, μM/g protein	2.2 (0.9)

Pg: picogram; g: gram; μM: micromole; IL-17A: interleukin 17A; IL-6: interleukin 6; IL-8: interleukin 8; IL-10: interleukin 10; MMP-2: matrix metalloproteinases 2; TBARS: substances reactive to thiobarbituric acid. Notes: Values expressed as median (interquartile range).

 Regarding aerobic fitness assessed by CPET, the mean peak VO_2_ was 35.4±4.6 mL·kg^−1^·min^−1^, while at VT_1_ it was 60.6±15.0% of peak values. The mean BR of the patients was 44.0±12.7%, indicating minimal ventilatory limitations. Complete data on CPET variables are presented in Table 4. 

**Table 4.** Cardiopulmonary exercise testing variables.

Variables		n=12
Rest		
	HR (bpm)	96.4±12.5
	SpO_2_ (%)	97.3±1.2
	VO_2_ (L·min^−1^)	0.4±0.1
	VO_2_ (mL·kg^−1^·min^−1^)	9.7±3.8
Ventilatory threshold		
	HR (bpm)	145.1±17.6
	SpO_2_ (%)	97.5±1.4
	VO_2_ (L·min^−1^)	0.9±0.3
	VO_2_ (mL·kg^−1^·min^−1^)	21.4±5.3
	VO_2_ (% of peak)	60.6±15.0
	V_E_ (L·min^−1^)	21.8±10.0
	V_E_/VO_2_	23.9±2.4
	V_E_/VCO_2_	25.4±1.8
	RER	0.9±0.05
Peak exercise		
	HR (bpm)	188.3±7.0
	SpO_2_ (%)	96.7±3.0
	VO_2_ (L·min^−1^)	1.5±0.4
	VO_2_ (mL·kg^−1^·min^−1^)	35.4±4.6
	V_E_ (L·min^−1^)	46.5±16.5
	V_E_/VO_2_	31.1±4.0
	V_E_/VCO_2_	28.2±2.9
	RER	1.1±0.07
	Breathing reserve (%)	44.0±12.7

HR: heart rate; SpO_2_: peripheral oxygen saturation; VO_2_: oxygen consumption; VCO_2_: carbon dioxide consumption; V_E_: minute volume; V_E_/VO_2_: ventilatory equivalent for oxygen consumption; V_E_/VCO_2_: ventilatory equivalent for carbon dioxide production; RER: respiratory exchange ratio; Bpm: beats per minute L: liters; mL: milliliters; kg: kilograms. Notes: Values expressed as mean±standard deviation.

**Figure 2:**
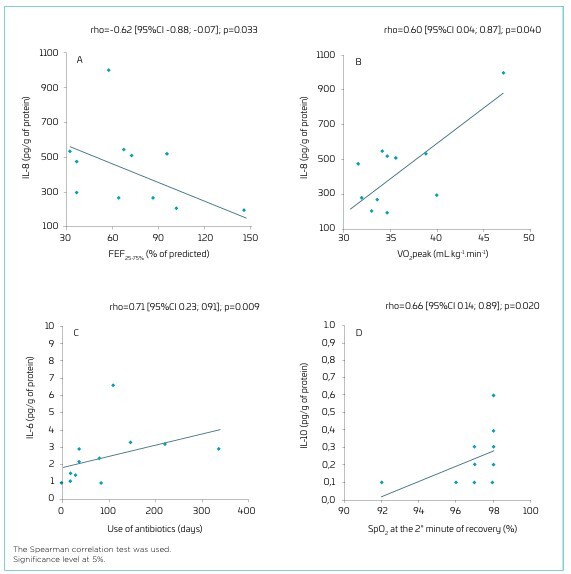
Correlation between interleukins, pulmonary function variables, and aerobic fitness. The graphs present the correlation between IL-8 and FEF_25–75%_ (A), IL-8 and VO2peak (B), IL-6 and days on antibiotic treatment (C), and IL-10 and SpO_2_ at the 2nd minute of recovery (D).

 Correlation analysis (Figure 2) between the inflammatory profile and key parameters of lung function, exercise capacity, and morbidity revealed a correlation between IL-6 concentration and the number of days on antibiotics (rho=0.71 [95% confidence interval (CI) 0.23, 0.91]; p=0.009), and an inverse moderate correlation between IL-8 and FEF_25–75%_ (rho=-0.62 [95%CI -0.88; -0.07]; p=0.033). In terms of aerobic fitness, a positive moderate correlation was found between IL-8 and VO_2_peak in mL·kg^−1^·min^−1^ (rho=0.60 [95%CI 0.04; 0.87]; p=0.040), and a positive moderate correlation was found between SpO_2_ at the second minute of recovery and IL-10 (rho=0.66 [95%CI 0.14; 0.89]; p=0.020). No relevant correlations were found between the studied variables and other cytokines, nor with MMP-2 or TBARS. 

## DISCUSSION

 The results presented here indicate that, when comparing IL-8 and TBARS concentrations in sputum samples from individuals with CF and in saliva from healthy controls, an increase was observed in individuals with CF. Moreover, increased inflammation (IL-8) correlates with worsening lung function, as reflected in FEF_25–75%_, a marker of small airway obstruction in CF patients. A correlation was also observed between inflammation, as represented by IL-6 concentration, and a higher number of days on antibiotics. 

 CF is characterized by a dysregulated inflammatory response, which can be recognized, as in our study, by the predominance of neutrophils and the broad expression of pro-inflammatory cytokines such as IL-8.^15-19^ Chronic inflammation and exacerbations lead to a progressive decline in lung function.^20^ IL-8 mediates neutrophil adhesion to the site of inflammation, leading to an intense, chronic neutrophilic inflammatory response in CF patients.^3,21,22^ Similar to the study by Sagel et al.,^23^ our study also demonstrated higher IL-8 concentrations among the cytokines analyzed in sputum. 

 Imbalances and typical changes in sputum favor bacterial colonization, with pulmonary exacerbations being one of the main contributors to significant morbidity in CF.^5,6^ A previous study indicated that IL-6 concentrations in patients undergoing treatment for exacerbations increased after antibiotic use.^24^ In our study, higher IL-6 levels correlated with a higher number of days on antibiotics. 

 Regarding oxidative stress, Oliveira et al.^25^ observed higher TBARS concentrations in individuals with bronchiectasis than in healthy individuals. Similarly, Benabdeslam et al.^26^ also found higher TBARS concentrations in CF patients compared to healthy controls. Negative correlations between FEV_1_ and IL-8 were observed in studies by Mayer-Hamblett et al.^27^ and Sagel et al.^28^ However, although the later study by Sagel et al.23 reported an association, we did not observe a significant correlation between FEV_1_ and IL-8 (rho=-0.524; p=0.08), possibly due to the limited sample size. On the other hand, as seen in the study by Sagel et al.,28 we found a strong and significant negative correlation between IL-8 and FEF_25-75%_. Although FEF_25-75%_ is not usually considered a primary or robust outcome for lung function assessment, the significant correlation observed may reflect early alterations in small airway obstruction in CF patients. 

 To the best of our knowledge, this is the first study to compare oxidative stress levels between CF patients and healthy controls and to report correlations between sputum inflammatory markers and key CPET variables, which are considered important prognostic tools in CF patients. Individuals with a VO_2_ less than 82% predicted have a higher risk of severe outcomes, as do those with a VO2peak less than 32 mL·kg^−1^·min^−1^.^29^ In our sample, 83.3% of individuals had values within the normal range for this variable, which may help to explain the positive correlation with IL-8. IL-10 plays a crucial role in reducing lung damage. However, CF is characterized by a limitation in the release of this anti-inflammatory cytokine.^30^ In our study, we observed a potential positive effect of this cytokine, with higher IL-10 levels correlating with better SpO_2_ during the exercise recovery period. 

 This study presents several limitations, including the limited number of patients and the use of banked control saliva samples. Although the sample size estimation indicates adequate power, these may represent an initial step toward further studying the importance of basal levels of inflammation in the sputum of non-exacerbated individuals with CF, and caution is needed when interpreting the findings, as it may affect their reliability and generalizability to other contexts or populations. The inclusion of samples from patients with preserved lung function and exercise capacity may have contributed to the results obtained. As for the study design, a limitation of correlational analyses is the potential for reverse causality, as the observed associations do not allow one to determine the direction of the relationship between variables. Therefore, further studies including patients with a broader clinical profile and under the effects of new CFTR modulator drugs are needed to confirm the findings of this study, as well as studies with a longitudinal or experimental design to investigate the causal direction of the identified relationships. 

 The results presented indicate that non-exacerbated individuals with CF exhibit higher basal sputum inflammatory levels than healthy saliva controls. IL-8 and IL-6 correlated with worsening pulmonary function and the number of days of antibiotic use. In addition, our data also suggest that the inflammatory process is not directly associated with aerobic fitness. 

## Data Availability

The database that originated the article is available with the corresponding author.
